# Distribution and Contamination Assessment of Soil Heavy Metals in the Jiulongjiang River Catchment, Southeast China

**DOI:** 10.3390/ijerph16234674

**Published:** 2019-11-23

**Authors:** Qian Zhang, Guilin Han, Man Liu, Xiaoqiang Li, Lingqing Wang, Bin Liang

**Affiliations:** 1 School of Water Resources and Environment, China University of Geosciences (Beijing), Beijing 100083, China; zhangqian9@cugb.edu.cn (Q.Z.); lman@cugb.edu.cn (M.L.); xiaoqli@cugb.edu.cn (X.L.); liangbin@cugb.edu.cn (B.L.); 2 Institute of Geographic Sciences and Natural Resources Research, Chinese Academy of Sciences, Beijing 100101, China; wanglq@igsnrr.ac.cn

**Keywords:** environmental pollution, heavy metal content, toxic risk assessment, Fujian province

## Abstract

A total of 63 soil samples were collected from three soil profiles (yellow soil, red loam, red soil) from Jiulongjiang river catchment to investigate the distribution, controlling factors, and toxic risks of heavy metals, including Cr, Mn, Fe, Cu, Zn, Cd, Pb, and Ni. The results showed that Cr and Cd in soils were enriched. The relationships between heavy metals and soil properties were assessed by principal component analysis. The results indicated that soil organic matter (SOM) played a fundamental role in controlling Cd and Pb in yellow soil and red loam sites. The Cd was significantly correlated with Pb and Cu, and Cr, Zn, Ni, Fe displayed strong correlations with each other, however, no statistical correlation was found between Cd and Cr. The enrichment factor and geoaccumulation index analyses showed that the soils in the study area were contaminated by Cd. Potential ecological risk analyses indicated that Cd posed a considerable ecological risk in yellow soils, and posed a moderate ecological risk in red loams and red soils.

## 1. Introduction

Heavy metals (HMs) have caused significant ecological environmental concern due to their toxicity and persistence. Moreover, heavy metal accumulation in soils is a serious potential threat to ecosystems [1,2]. Heavy metal pollution not only impairs soil’s chemical/physical properties and leads to soil nutrient loss, but also affects the soil organisms and contaminates the food chain. Organisms in soil have the ability to accumulate HMs and finally to pose a threat to human health, such as hypophosphatemia, neurotoxicity, liver damage, and heart disease [3]. Examples of heavy metals include Pb, Cd. Tang et al. reported that Pb and Cd are two of the most common toxic HMs which are related to cancers and malfunction of the nervous system [4]. As a nonessential metal, Cd has been classified as a human carcinogen [5], and Cd can enter the brain parenchyma and neurons and lead to neurological alterations, and finally result in memory deficits, attention deficits, and olfactory dysfunction [6]. By studying the data on the national communique of soil pollution survey of China in 2014, soils are significantly polluted in some regions, and the quality of agricultural land soil is particularly concerning [7]. The rapid development of industrialization and the increasing application of agrochemicals have led to the accumulation of HMs in soils [8,9,10]. High concentrations of HMs in soils may lead to ecological damage and threaten the health of humans and animals [11,12,13,14,15]. 

Natural and anthropogenic sources are considered the two main sources of HMs in soils [16]. HM contents in soils are natural components of the Earth’s crust and mainly depend on the geological parent material [17]. Meanwhile, anthropogenic sources, such as industrial activities, agricultural practices, and vehicle exhaust, can also increase the contents of HMs [18,19,20]. In urban areas, HMs may originate from various sources, such as industrial activities, mining, smelting, power generation, fossil fuel combustion, and waste disposal [21,22,23]. Anthropogenic input of HMs into soils may cause the deterioration of soil function and change soil’s physical/chemical properties, which might create other environmental problems [24]. The assessment of ecological risk from HM contamination in soil has gained more attention in recent studies [25].

The Jiulongjiang river catchment is located in Fujian province, southeast China, and it plays an important role in the development of economy, especially in agricultural economy. In recent years, problems with pollution in Jiulongjiang river and nearby catchments have arised [26,27,28]. The environmental problems in Jiulongjiang river catchment are concluded as follows. Firstly, there are abundant mineral resources, including iron ore and coal mine, in this area, and the main environmental problem is caused by mining and dumping of discarded slag [29]. Secondly, the applications of chemical fertilizers and nutrients in agricultural activities as the anthropogenic inputs may result in the accumulation of HMs in soils. Finally, soil erosion caused by man-made mountain development and reforestation has resulted in the reduction of soil fertility and cultivated land erosion [30,31], and these problems directly or indirectly lead to soil heavy metal pollution and reduced environmental quality.

HM concentrations in soils are also related to a series of local environmental factors, including soil types and physicochemical properties. Many studies have researched the relationships between heavy metal concentrations and soil properties, such as soil organic matter (SOM) and pH [32]. Assessment of soil properties and research on the influence of soil properties on heavy metals are important for characterizing soil HM distributions and tracing the migration of HMs in soils. Studies on HM distribution and assessing the ecological risks will help managers make strategic decisions to arrange reasonable industrial and agricultural activities and prevent risks to human health and environment. Until now, only a few studies have focused on vertical distribution and ecological risk of HMs in soil profile at Jiulongjiang river catchment in Southeast China [26].

In this study, eight selected HMs (Cr, Mn, Fe, Cu, Zn, Cd, Pb, and Ni) in 63 soil samples collected from three soil profiles in Jiulongjiang river catchment were studied with objectives: (1) To determine the vertical distribution of HMs in soils, (2) to research the relationships between soil HMs and soil properties, (3) to assess the HM toxic risks using enrichment factor (EF), geoaccumulation index (I_geo_), and potential ecological risk index (R_I_).

## 2. Materials and Methods

### 2.1. Study Area

**Figure 1:**
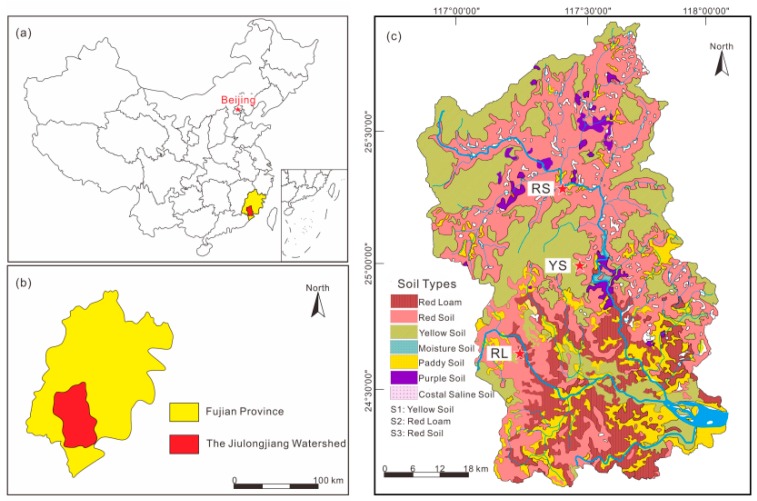
Distributions of soil types and sampling sites in the Jiulongjiang River catchment.

The study sites were located in Jiulong river catchment (24°13′–25°51′ N, 116°47′–118°02′ E), Fujian province, Southwest China (Figure 1). The study area is controlled by the subtropical oceanic monsoon climate. The average annual temperature is 21 °C and the mean annual precipitation is 1200–2000 mm [33]. The forest coverage accounts for over 60% with varied vegetation. In this catchment, soil is characterized by red loam, red soil, yellow soil, and paddy soil. The area proportion of red loam is about 62%, and red soil is about 16% [34]. 

### 2.2. Sampling and Analysis

The sampling sites were selected from yellow soil (YS) at abandoned agricultural land, red loam (RL), and red soil (RS) at forest land along Jiulongjiang river catchment in January 2018. Soil profiles of a depth of 100 cm were selected to research the vertical distribution of HMs, because the variation extent of HM contents focuses on this soil layer. A total of 63 soil samples were collected from the three soil profiles, and each profile was cut into 5 cm sections, and the description of sampling sites can be seen in Table 1. Soil samples were air-dried at 25 °C and sieved through a 2 mm nylon sift to remove coarse debris. Then, soil samples were ground until all the particles would pass through a 200-mesh sift. The soils were digested with HNO_3_-HF-HClO_4_, and the concentrations of eight heavy metals (i.e., Cr, Mn, Fe, Cu, Zn, Cd, Pb, and Ni) in soils were determined using ICP-MS (Elan DRC-e, Perkin Elmer). Soil samples, reagent blanks, and standard reference samples were synchronously analyzed. Total phosphorus (TP), soil particle distribution, and pH were measured following the methods as in our report [35], and soil organic carbon (SOC) and soil organic nitrogen (SON) were measured following the methods reported by Liu [36,37].

### 2.3. Data Analysis

In the present study, the degrees of HM contamination in soils were assessed using enrichment factor (EF) [38] and geo-accumulation (I_geo_) [39], and potential ecological risk index (R_I_) [40] values were selected to evaluate potential ecological risks.

EF is defined as follows:

EF = (C_m_/C_Al_)/(B_m_/C_Al_), (1)

where C_m_ is the measured value of target element in soils, and B_m_ is the background value (BV) of this element, and the reference background content was obtained from the Fujian soils in this study [41]. Aluminum (Al) was used as the geochemical normalizing element.

I_geo_ is calculated by the following formula:

I_geo_ = log_2_(C_m_/1.5B_m_), (2)

where C_m_ and B_m_ are the determined value and background value of target element in soils, respectively. In this study, B_n_ denoted the content of heavy metals in the soils of Fujian province [41]. 

The calculation equation of R_I_ is given as follows:

(3) $$R_{I}=\sum Er_{m}=\sum T_{m}C_{f}=\sum T_{m}(C_{m}/B_{m}),$$

where Er_m_ is the monomial potential ecological risk factor, and C_f_ is the contamination factor, and T_m_ is the biological toxicity factor (i.e., Cd = 30, Cr = 2, Cu = 5, Pb = 5, Ni = 5 and Zn = 1) [40]. 

## 3. Results and Discussion

### 3.1. Soil Properties and Heavy Metal Concentrations

**Figure 2:**
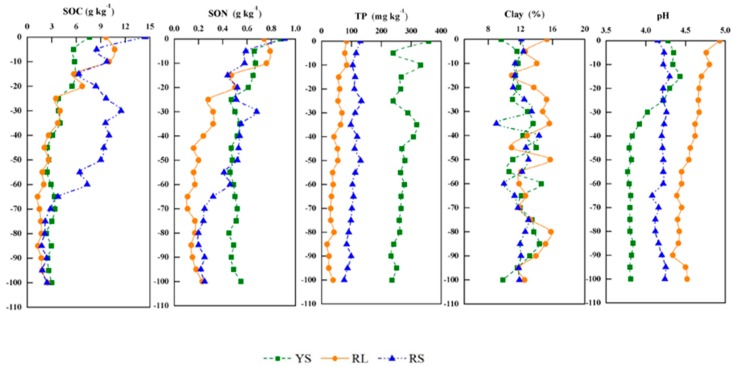
Vertical distribution of soil properties, including soil organic carbon (SOC), soil organic nitrogen (SON), pH, total phosphorus (TP) and clay contents, at the three sampling sites.

The natural concentration of HMs in soils largely depends on the parent materials, and its distribution is also influenced by soil properties [42]. Vertical distribution of soil properties, including SOC, SON, pH, TP and clay contents, in the three types soils (red loam, red soil, and yellow soil) are shown in Figure 2. The pH in soil profiles revealed strong acid with values ranging from 3.77 to 4.96, and pH at 30 cm depth decreased greatly in site YS, implying a likely influence of human activity. SOC contents, which ranged from 1.28 to 14.42 g kg^−1^, decreased with the increase of soil depth with obvious change trends in upper 30 cm of profiles. SON contents decreased with the increase of soil depth with a range of 0.88–0.11 g kg^−1^. Clay content ranged from 9.60% to 15.78% without obvious differences among the three sampling sites. TP contents at site YS were much higher than that at sites RL and RS, which was likely related to the previous application of fertilizer in abandoned agricultural land at site YS.

The concentrations of HMs (Cr, Mn, Fe, Cu, Zn, Cd, Pb, and Ni) in soils are presented in Table A1. Compared with the references of BV in Fujian soils [41], the Cr and Cd concentrations in all the profile soils were much higher than BV, and the Mn, Fe, Cu, Zn, Pb, and Ni concentrations were much lower than BV. The distributions of HMs in topsoils were quite different at three sampling sites. Cu, Cd, and Pb contents were the highest at site YS, which was associated with human activities [43]. Zn and Mn contents were the highest at site RL. Cr, Fe, and Ni contents were the highest at site RS and were the highest in red soil, which may depend on soil types [41].

### 3.2. Vertical Profiles of Heavy Metals

**Figure 3:**
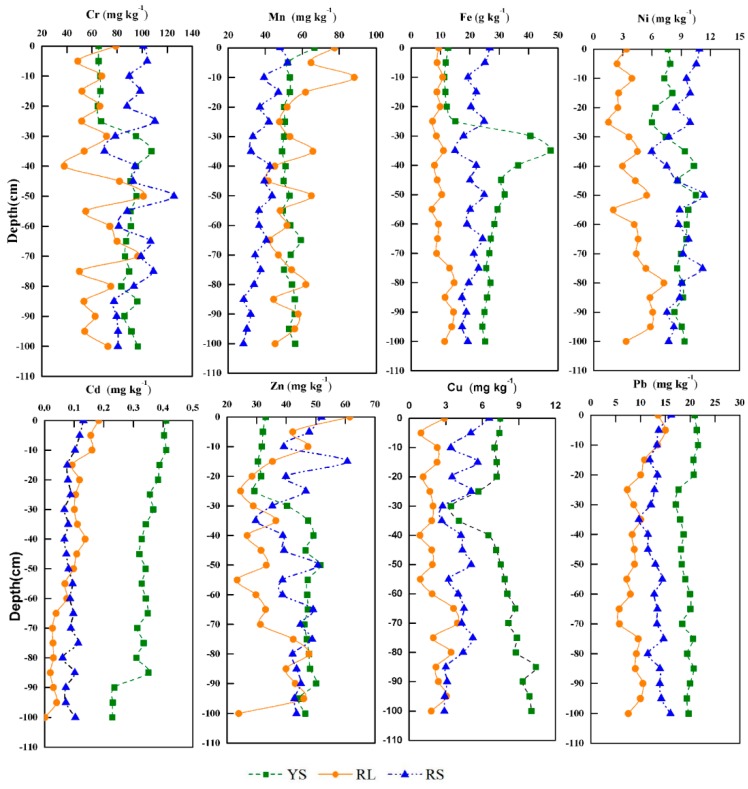
Distributions of heavy metals (HMs) in profile soils at the three sampling sites.

Distributions of HMs, including Cr, Mn, Fe, Cu, Zn, Cd, Pb, and Ni, along soil profiles at the three sampling sites are shown in Figure 3. At site YS, the Cr, Fe, Ni, Cu, and Zn contents varied remarkably at 25–35 cm soil layer. Cd, Pb, and Mn content decreased with soil depth in the upper 30 cm, while obvious changes were not observed in the soils lower than 30 cm, which might be related to previous agricultural activities, such as plowing and fertilization [44]. The contents of most HMs (e.g., Cr, Cu, Zn, Pb, and Ni) at site RL showed an irregular variation and fluctuated remarkably, which might be influenced by soil properties. At site RS, the contents of all the eight HMs were the highest in topsoils and decreased slightly with the increase of soil depth, which might be affected by atmospheric deposition related to the combustion of coal and mining activities in Beixi region [31].

### 3.3. Pearson Correlation Analysis

Pearson correlation analysis was used to identify the relationship between HM concentrations and soil properties in different soil types (Table 2), such as yellow soil (YS), red loam (RL), red soil (RS). As shown in site YS, positive correlations were found between Cd and TP, pH, SON, and SOC. Pb also had significantly positive correlations with SON and SOC (*p* < 0.01). However, Cr, Fe, and Zn had negative correlations with pH, SON, and SOC (*p* < 0.01). Ni had a negative correlation with SOC. One potential reason may be the adsorption of HMs by SOM, which has a greatly absorptive capacity for metals, such as Cd and Pb [45]. However, humic acid and humin may reduce the contents of some metals in soils [46], so the higher organic matter contents may not immobilize more metals, such as Cr, Fe, and Zn. In accordance with the results from our previous reports, Cd have a strong positive correlation with SOC, while Cr and Fe have negative relationships with SOC in karst soils from southwest China [35]. TP and pH showed a positive correlation with SON and SOC in soils at YS and RS sites. At site RL, Mn, Cd, and Pb maintained a remarkable correlation with TP, pH, SON, and SOC, and negative correlations were found between Ni and TP, pH, SON and SOC. These results can be also explained as the influence of SOM. At site RS, the relationships between the HMs and soil properties, including SOC, SON, TP, pH, and clay, were not close and only Mn showed correlations with TP, SON, and SOC. A weak positive correlation was found between clay with Fe and Zn in yellow soil, and no correlation was observed between clay and HMs in other soil types.

Correlation relationships among HMs were determined using Pearson correlation analysis to provide information on their sources and transport [47]. The results of Pearson correlation analysis in soils at all sampling sites are displayed in Table 3. Cd was significantly correlated with Pb and Cu (*p* < 0.01), indicating that similar geochemical behavior or input sources are likely related to the use of pesticides and fertilizers. Cr, Zn and Ni, Fe displayed a strongly positive correlation (*p* < 0.01) with each other, suggesting the possibility of their common origin. However, no statistical correlation was found between Cd and Cr, likely indicating the different origins of these two elements.

### 3.4. Contamination Assessment of Heavy Metals

**Figure 4:**
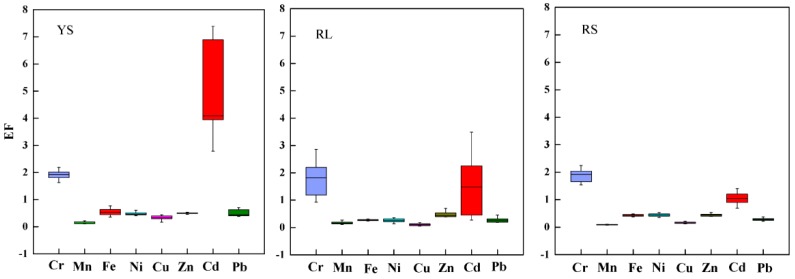
Enrichment factor (EF) of HMs at the three sampling sites.

The EF values of selected HMs in soil profiles are depicted in Figure 4. The EF values of most HMs, including Mn, Cu, Zn, Fe, Pb, and Ni, were less than 1.0, and most of them were less than 0.5 at three sampling sites. The EF values of Cr and Cd were much higher than that of other HMs. The Cr was at minimal enrichment levels (1 < EF < 2.0) in most soils without significant changes in EF values along the profiles. The Cd was at significant enrichment level (EF > 5.0) in soils above 30 cm layer at YS site, indicating strong anthropogenic sources, including agrochemicals and chemical fertilizers [48]. Cd was at moderate enrichment level (2.0 < EF < 5.0) in soils above 30 cm layer at RL site, while was at minimal enrichment level (0.5 < EF < 1.5) at RS site, which might be influenced by industrial activities, including mining and fossil fuel, near RL site.

**Figure 5:**
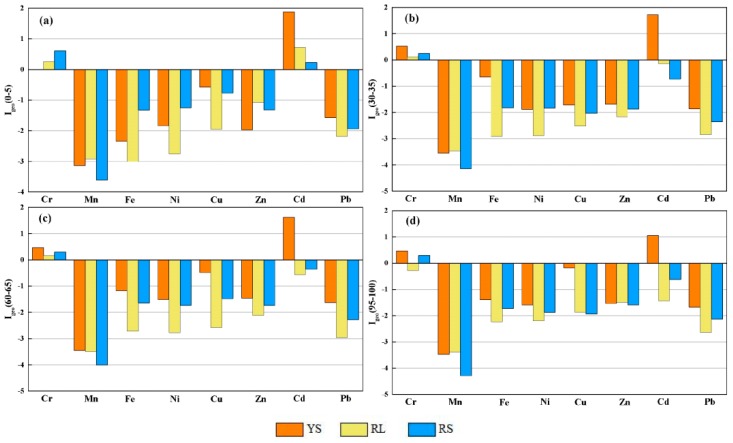
Geo-accumulation indexes (I_geo_) of HMs at 0–5 cm depth (**a**), 30–35 cm depth (**b**), 60–65 cm depth (**c**) and 95–100 cm depth (**d**) at the three sampling sites.

Figure 5 demonstrates the I_geo_ values of HMs in various layers, including 0–5, 30–35, 60–65, and 95–100 cm depth, which can represent the change of these HMs in the whole vertical profile. I_geo_ values of Mn, Cu, Zn, Fe, Pb, and Ni in all soil profiles were less than 0, suggesting that soils in the study area were uncontaminated by these HMs. I_geo_ values of Cr were lower than 1.0 at the four depths without a significant decrease with the increase of soil depth. Combining with the results of EF analysis, the results indicated the soils were not contaminated by anthropogenic inputs, and Cr enrichment in soils mainly depended on bedrocks [2]. I_geo_ values of Cd were higher than 1.0 in all soils at YS site, while ranged from 0 to 1 in topsoils at RL and RS sites, indicating that Cd pollution level at YS site was more serious than at RL and RS sites, and only topsoils at RL and RS sites were contaminated by Cd.

### 3.5. Assessment of Potential Ecological Risk

Potential ecological risk index (R_I_) was comprehensively introduced to assess the potential ecological risks caused by HMs, which was considered to be applied in various study domains, such as ecological environment, biological toxicology, and environmental chemistry [40,49]. The results of R_I_ and Er_m_ for Cr, Cu, Zn, Cd, Pb, and Ni in the soils at 0, 30, 60, and 100 cm depth in the three sites are listed in Table 4. Except for Cd, the Er_m_ values of most HMs (<40) decreased as follows: Cd > Cr > Pb > Ni > Cu > Zn, indicating the pollution degree of these HMs. The highest Er_m_ for Cd were found at site YS, which had a considerable ecological risk of Cd (80 ≤ Er_m_ < 160) at 0–100 cm depth according to the description of risk classification [20]. Cd posed a moderate ecological risk (40 ≤ Er_m_ < 80) in topsoils at sites of RL and RS. The R_I_ values exhibited a moderate ecological risk (95 ≤ R_I_ < 190) at site YS, and low ecological risk (R_I_ < 95) at sites of RL and RS, which were associated with the degree of anthropogenic disturbance. The type of land-use at YS site was abandoned agricultural land, which used to be tea plantation several years ago. The sources of Cd pollution of this site were influenced by previous agricultural activities, including fertilizer and pesticide. The accumulation of Cd in topsoils at site RL and RS presumably results from the effects of atmospheric HM deposition from mining and fossil fuel exploitation.

## 4. Conclusions

This study demonstrates the distribution and influencing factors of eight selected HMs, including Mn, Fe, Cu, Zn, Pb, and Ni, in soil profiles in Jiulongjiang river catchment and the conclusions are summarized as follows.

1. The contents of these eight HMs were much lower than local reference background values, whereas Cr and Cd were enriched. Cr enrichment in soils was mainly dependent on local bedrocks.
2. The Cd, Pb and Cu were significantly correlated (*p* < 0.01), indicating similar geochemical behavior or input anthropogenic sources likely related to the use of pesticides and fertilizers. The Cr, Zn, Ni, and Fe displayed strongly positive correlation (*p* < 0.01) with each other, suggesting the common origin of HMs with a natural source. However, the statistical correlation between Cd and Cr was not found.
3. EF and I_geo_ analysis indicated that soils were contaminated by Cd in abandoned agricultural land (YS), and pollution degree in abandoned agricultural land was more serious than that in forest lands (RL and RS).
4. Potential ecological risk analysis indicated that Cd posed a considerable ecological risk in all profile soils at site YS, and posed a moderate ecological risk in topsoils under at RL and RS sites. Agricultural activities, including fertilization and pesticides, were the main input of Cd at YS site, and industrial activities, including mining and fossil fuel, were the contributors of Cd at RL and RS sites. Although soils in the Jiulongjiang River catchment were only contaminated by Cd, migration and enrichment of other metals should be controlled by arranging reasonable industrial and agricultural activities.

## Figures and Tables

**Table 1:** Description of sampling sites in study area.

Sampling Site	Depth (cm)	Land-Use Types	Soil Types	Visible Characteristics
YS	100	Abandoned agricultural land (tea plantation had been abandoned and covered by weed)	Yellow soil	0–30 cm, gray humus layer, fine sand, loose 30–50 cm, gray mixed red, fine sand, loose 50–100 cm, yellow, clay-grained, tight
RL	100	Forest land	Red loam	0–40 cm, dark brown humus layer, mixed coarse sand, loose 40–70 cm, gray, mixed coarse sand, loose 70–100 cm, red, mixed coarse sand, loose
RS	100	Forest land	Red soil	0–65 cm, gray red humus layer, fine-grained, loose 65–100 cm, red, fine particles, loose

**Table 2:** Pearson correlation between heavy metals and soil properties.

Sampling Site		Cr	Mn	Fe	Cu	Zn	Cd	Pb
YS	TP	−0.081	0.269	0.105	−0.492 *	−0.177	0.516 *	0.030
	pH	−0.848 **	0.103	−0.712 **	−0.376	−0.953 **	0.714 **	0.427
	SON	−0.639 **	0.553 **	−0.587 **	−0.121	−0.657 **	0.587 **	0.578 **
	SOC	−0.747 **	0.375	−0.624 **	−0.318	−0.830 **	0.731 **	0.502 *
	Clay	0.422	−0.330	0.450 *	−0.012	0.434 *	−0.075	−0.104
RL	TP	0.060	0.677 **	−0.375	−0.267	0.246	0.879 **	0.653 **
	pH	−0.101	0.603 **	−0.420	−0.267	0.274	0.886 **	0.662 **
	SON	−0.204	0.738 **	−0.184	−0.286	0.448 *	0.802 **	0.869 **
	SOC	−0.121	0.726**	−0.232	−0.240	0.428	0.803 **	0.842 **
	Clay	0.083	0.391	0.264	0.045	0.314	0.158	0.174
RS	TP	0.630 **	0.790 **	0.701 **	0.627 **	0.273	0.088	−0.123
	pH	−0.300	0.047	−0.289	−0.269	−0.107	−0.294	−0.200
	SON	0.151	0.624 **	0.363	0.364	−0.059	0.233	−0.091
	SOC	0.153	0.583 **	0.312	0.307	−0.152	0.110	−0.220
	Clay	0.422	0.210	0.396	0.250	0.201	−0.094	0.154

** *p* < 0.01; * *p* < 0.05.

**Table 3:** Pearson correlation among HMs.

	Cr	Mn	Fe	Cu	Zn	Cd	Pb	Ni
Cr	1							
Mn	−0.267 *	1						
Fe	0.697 **	−0.158	1					
Cu	0.436 **	0.130	0.555 **	1				
Zn	0.553 **	0.141	0.617 **	0.428 **	1			
Cd	0.083	0.336 **	0.432 **	0.719 **	0.118	1		
Pb	0.262 *	0.164	0.565 **	0.833 **	0.390 **	0.877 **	1	
Ni	0.759 **	−0.326 **	0.774 **	0.666 **	0.641 **	0.312 *	0.593 **	1

** *p* < 0.01; * *p* < 0.05.

**Table 4:** Heavy metal potential ecological risk indexes in the Jiulongjiang River catchment.

Sampling Site	Depth (cm)	Er						R_I_
		Cr	Cu	Zn	Cd	Pb	Ni	
YS	0	2.97	1.64	0.38	166.17	2.53	2.10	175.79
	30	4.32	0.75	0.47	148.64	2.07	2.03	158.28
	60	4.14	1.77	0.55	138.55	2.42	2.63	150.05
	100	4.39	2.20	0.54	92.84	2.38	2.57	104.92
RL	0	3.59	0.63	0.71	74.29	1.64	0.93	81.81
	30	3.26	0.43	0.34	40.76	1.04	1.00	46.83
	60	3.37	0.41	0.35	30.42	0.96	1.15	36.66
	100	2.46	0.67	0.53	16.54	1.21	1.60	23.01
RS	0	4.59	1.44	0.60	52.93	1.96	2.99	64.51
	30	3.58	0.60	0.41	27.22	1.46	2.12	35.39
	60	3.70	0.88	0.45	34.95	1.55	2.40	43.93
	100	3.67	0.63	0.51	41.97	1.94	2.12	50.83

## Appendix A

**Table A1:** Heavy metal contents in soils measurement and reference values.

Sampling Site	Depth (cm)	Cr (mg kg^−1^)	Mn (mg kg^−1^)	Fe (g kg^−1^)	Cu (mg kg^−1^)	Zn (mg kg^−1^)	Cd (mg kg^−1^)	Pb (mg kg^−1^)	Ni (mg kg^−1^)
YS	0	65.4	66.57	12.59	7.470	33.07	0.410	20.91	7.627
	5	65.22	52.24	11.86	7.387	32.15	0.403	21.34	7.864
	10	66.52	53.3	11.31	7.002	31.84	0.410	21.60	7.275
	15	66.7	53.33	11.71	7.184	30.46	0.388	20.63	8.093
	20	64.82	50.17	12.13	7.177	31.55	0.383	20.72	6.368
	25	67.16	50.7	15.11	5.681	29.28	0.355	17.66	5.973
	30	95.08	50.12	40.6	3.418	40.31	0.367	17.10	7.401
	35	107.5	49.14	47.49	4.091	47.4	0.342	17.99	9.38
	40	95.02	50.96	36.4	6.511	49.25	0.328	18.68	10.30
	45	90.77	49.95	30.62	7.135	46.57	0.320	18.12	8.652
	50	95.45	53.15	31.89	7.505	51.54	0.341	18.33	10.50
	55	90.87	49.48	29.46	7.840	47.29	0.328	18.99	9.729
	60	91.13	53.63	28.34	8.055	47.14	0.342	19.99	9.561
	65	87.22	59.16	27.15	8.704	47.36	0.348	20.02	9.521
	70	86.45	53.78	26.64	8.139	46.19	0.313	18.41	8.995
	75	89.75	50.12	25.56	8.845	47.02	0.335	20.59	8.584
	80	83.47	54.47	27.03	8.751	47.69	0.310	19.40	9.103
	85	96.2	55.94	25.88	10.41	48.09	0.351	20.73	9.187
	90	85.88	55.89	24.97	9.333	50.11	0.236	19.98	8.319
	95	91.4	52.85	24.28	9.863	44.26	0.230	19.33	9.056
	100	96.52	56.27	25.22	10.04	46.48	0.229	19.68	9.346
RL	0	78.98	77.47	9.368	2.892	61.49	0.183	13.53	3.4
	5	48.66	64.72	8.855	0.9683	42.21	0.156	15.00	2.419
	10	67.8	88.06	10.75	2.302	47.34	0.160	13.44	3.927
	15	52.04	61.74	8.797	2.288	35.37	0.093	10.81	2.581
	20	66.32	51.85	9.893	1.145	28.46	0.118	10.01	2.499
	25	51.82	47.69	7.282	1.699	24.62	0.105	7.35	1.544
	30	71.72	53.3	8.612	1.959	28.9	0.101	8.63	3.637
	35	53.75	65.76	11.03	1.849	36.52	0.111	10.03	4.509
	40	37.85	45.14	7.986	0.8869	26.82	0.137	8.33	2.976
	45	81.9	41.65	8.871	1.85	31.51	0.109	8.74	4.305
	50	101	64.87	10.42	1.898	33.29	0.098	8.79	5.453
	55	55.04	48.05	7.115	0.912	23.46	0.069	7.25	2.034
	60	74.22	51.8	9.315	1.882	29.81	0.075	7.96	4.18
	65	80.03	42.56	8.99	3.64	32.99	0.039	5.71	4.582
	70	96.61	47.12	8.684	3.957	31.3	0.027	5.75	4.396
	75	49.93	54.23	13.09	1.982	42.42	0.029	9.54	5.376
	80	74.97	61.93	14.66	3.435	47.73	0.030	9.17	7.218
	85	53.59	44.52	11.56	2.196	39.97	0.020	8.95	5.798
	90	62.52	57.78	14.43	2.400	43.06	0.030	10.48	6.079
	95	54.22	55.91	13.8	3.062	45.95	0.041	9.96	5.835
	100	72.77	45.35	11.42	1.812	23.98	0.002	7.55	3.343
RS	0	101	48.01	26.78	6.573	51.86	0.131	16.19	10.87
	5	104	52.03	25.13	5.061	47.78	0.119	13.67	10.55
	10	89.82	39.33	19.37	3.417	39.2	0.103	13.30	9.547
	15	98.44	47.04	22.2	5.622	60.69	0.077	11.87	9.916
	20	88.02	37.13	20.39	3.525	39.92	0.079	13.41	8.464
	25	110.2	41.95	24.87	5.088	46.64	0.088	12.81	9.903
	30	78.65	33.33	17.9	2.743	35.33	0.067	12.08	7.725
	35	69.99	32.27	14.96	2.676	29.71	0.080	9.66	5.979
	40	94.35	42.52	22.16	4.245	38.88	0.067	11.49	7.512
	45	92.8	39.54	20.09	4.365	39.28	0.074	11.50	8.604
	50	125.4	43.62	24.9	5.084	50.69	0.081	13.00	11.35
	55	88.15	36.66	20.1	3.242	38.79	0.095	14.37	8.834
	60	81.32	36.68	19.02	4.025	38.74	0.086	12.78	8.746
	65	106.7	40.63	24.33	4.499	49.11	0.097	13.41	9.762
	70	99.08	34.57	21.34	4.311	44.82	0.089	13.32	9.176
	75	109	37.53	22.97	5.221	48.77	0.114	14.64	11.19
	80	93.45	33.93	19.66	4.434	42.18	0.061	11.48	9.055
	85	77.56	28.39	17.33	3.008	43.54	0.102	13.91	8.848
	90	79.63	32.14	18.79	3.111	44.99	0.072	13.90	7.541
	95	80.77	30.07	17.34	2.924	42.78	0.072	14.17	8.25
	100	80.69	28.22	19.32	2.884	43.5	0.104	16.04	7.71
Background value [41]		14	391	42.4	22.8	96.1	0.074	41.3	18.2

## References

[1] Zhang T., Xu W., Lin X., Yan H., Ma M., He Z. (2019). Assessment of heavy metals pollution of soybean grains in North Anhui of China. Sci. Total Environ..

[2] Islam S., Ahmed K., Habibullah A.M., Masunaga S. (2015). Potential ecological risk of hazardous elements in different land-use urban soils of Bangladesh. Sci. Total Environ..

[3] Zoeteman B.C.J., Greef E.D., Brinkmann F.J.J. (1981). Persistency of organic contaminants in groundwater, lessons from soil pollution incidents in the Netherlands. Sci. Total Environ..

[4] Tang B., Tong P., Xue K.S., Williams P.L., Wang J.S., Tang L.L. (2019). High-throughput assessment of toxic effects of metal mixtures of cadmium (Cd), lead (Pb), and manganese (Mn) in nematode Caenorhabditis elegans. Chemosphere.

[5] Kobayashi E., Okubo Y., Suwazono Y., Kido T., Nishijo M., Nakagawa H., Nogawa K. (2002). Association between total cadmium intake calculated from the cadmium concentration in household rice and mortality among inhabitants of the cadmium-polluted Jinzu River basin of Japan. Toxicol. Lett..

[6] Antonio M.T., Corredor L., Leret M.L. (2003). Study of the activity of several brain enzymes like markers of the neurotoxicity induced by perinatal exposure to lead and/or cadmium. Toxicol. Lett..

[7] MEP of China (Ministry of Environmental Protection of China) (2014). National Soil Pollution Survey Bulletin. http://www.zhb.gov.cn/gkml/hbb/qt/201404/t20140417_270670.htm.

[8] Yang Q., Li Z., Lu X., Duan Q., Huang L., Bi J. (2018). A review of soil heavy metal pollution from industrial and agricultural regions in China: Pollution and risk assessment. Sci. Total Environ..

[9] Wu W., Wu P., Yang F., Sun D., Zhang D.X., Zhou Y.K. (2018). Assessment of heavy metal pollution and human health risks in urban soils around an electronics manufacturing facility. Sci. Total Environ..

[10] Liu J., Liu Y.J., Liu Y., Liu Z., Zhang A. (2018). Quantitative contributions of the major sources of heavy metals in soils to ecosystem and human health risks: A case study of Yulin, China. Ecotoxicol. Environ. Saf..

[11] Yadav I.C., Devi N.L., Singh V.K., Li J., Zhang G. (2019). Spatial distribution, source analysis, and health risk assessment of heavy metals contamination in house dust and surface soil from four major cities of Nepal. Chemosphere.

[12] Huang J., Li F., Zeng G., Liu W., Huang X., Xiao Z., He Y. (2016). Integrating hierarchical bioavailability and population distribution into potential eco-risk assessment of heavy metals in road dust: A case study in Xiandao District, Changsha city, China. Sci. Total Environ..

[13] Rahman M.S., Khan M.D.H., Jolly Y.N., Kabir J., Akter S., Salam A. (2019). Assessing risk to human health for heavy metal contamination through street dust in the Southeast Asian Megacity: Dhaka, Bangladesh. Sci. Total Environ..

[14] Liu J., Liang J., Yuan X., Zeng G., Yuan Y., Wu H., Huang X., Liu J., Hua S., Li F. (2015). An integrated model for assessing heavy metal exposure risk to migratory birds in wetland ecosystem: A case study in Dongting Lake Wetland, China. Chemosphere.

[15] Han G., Tang Y., Li F. (2017). Organic matter impact on distribution of rare earth elements in soil under different land uses. Clean-Soil Air Water.

[16] Hu Y., Cheng H. (2016). A method for apportionment of natural and anthropogenic contributions to heavy metal loadings in the surface soils across large-scale regions. Environ. Pollut..

[17] Liang J., Feng C., Zeng G., Gao X., Zhong M., Li X., Li X., He X., Fang Y. (2017). Spatial distribution and source identification of heavy metals in surface soils in a typical coal mine city, Lianyuan, China. Environ. Pollut..

[18] Xiao R., Wang S., Li R., Wang J.J., Zhang Z. (2017). Soil heavy metal contamination and health risks associated with artisanal gold mining in Tongguan, Shaanxi, China. Ecotoxicol. Environ. Saf..

[19] Liang X., Song J., Duan L., Yuan H., Li X., Li N., Qu B., Wang Q., Xing J. (2018). Source identification and risk assessment based on fractionation of heavy metals in surface sediments of Jiaozhou Bay, China. Mar. Pollut. Bull..

[20] Yi Y., Yang Z., Zhang S. (2011). Ecological risk assessment of heavy metals in sediment and human health risk assessment of heavy metals in fishes in the middle and lower reaches of the Yangtze River basin. Environ. Pollut..

[21] Karim Z., Qureshi B.A., Mumtaz M., Qureshi S. (2014). Heavy metal content in urban soils as an indicator of anthropogenic and natural influences on landscape of Karachi—A multivariate spatio-temporal analysis. Ecol. Indic..

[22] Martín J.A.R., Gutiérrez C., Escuer M., García-González M.T., Campos-Herrera R., Águila N. (2014). Effect of mine tailing on the spatial variability of soil nematodes from lead pollution in La Union (Spain). Sci. Total Environ..

[23] Islam M.S., Anmed M.K., Almamun M.H., Islam S.M.A. (2019). Sources and Ecological Risk of Heavy Metals in Soils of Different Land Uses in Bangladesh. Pedosphere.

[24] Vareda J.P., Valente A.J.M., Durães L. (2019). Assessment of heavy metal pollution from anthropogenic activities and remediation strategies: A review. J. Environ. Manag..

[25] Enya O., Lin C., Qin J. (2019). Heavy metal contamination status in soil-plant system in the Upper Mersey Estuarine Floodplain, Northwest England. Mar. Pollut. Bull..

[26] Lin C., Yu R., Hu G., Yang Q., Wang X. (2016). Contamination and isotopic composition of Pb and Sr in offshore surface sediments from Jiulong River, Southeast China. Environ. Pollut..

[27] Lin C., Hu G., Yu R., Yang Q., Yu W. (2016). Pollution assessment and source analysis of heavy metals in offshore surface sediments from Jiulong River. China Environ. Sci..

[28] Liu J., Han G., Liu X., Yang K., Li X., Liu M. (2019). Examining the Distribution and Variation of Dissolved Carbon Species and Seasonal Carbon Exports within the Jiulongjiang River Basin (Southeast China). J. Coast. Res..

[29] Wang W., Chen H., Chen M., Lin W., Lin Q., Ding G., Gong J. (2002). Ecological Environment Analysis of Soil and Water Conservation in Jiulong River Watershed. Res. Soil Water Conserv..

[30] Li X., Han G., Liu M., Yang K., Liu J. (2019). Hydro-geochemistry of the river water in the Jiulongjiang River basin, Southeast China: Implications of anthropogenic inputs and chemical weathering. Int. J. Environ. Res. Public Health.

[31] Liang B., Han G., Liu M., Yang K., Li X., Liu J. (2018). Distribution, sources, and water quality assessment of dissolved heavy metals in the Jiulongjiang River water, Southeast China. Int. J. Environ. Res. Public Health.

[32] Li C., Sun G., Wu Z., Zhong H., Wang R., Liu X., Guo Z., Cheng J. (2019). Soil physiochemical properties and landscape patterns control trace metal contamination at the urban-rural interface in southern China. Environ. Pollut..

[33] Yang K., Han G., Liu M., Li X., Liu J., Zhang Q. (2018). Spatial and seasonal variation of O and H isotopes in the Jiulong River, Southeast China. Water.

[34] Huang J., Li Q., Hong H., Lin J., Qu M. (2011). Preliminary study on linking land use/landscape pattern and water quality in the Jiulong River watershed. Environ. Sci..

[35] Zhang Q., Han G., Liu M., Liang T. (2019). Spatial distribution and controlling factors of heavy metals in soils from Puding Karst Critical Zone Observatory, southwest China. Environ. Earth Sci..

[36] Liu M., Han G., Zhang Q., Song Z. (2019). Variations and Indications of δ^13^C_SOC_ and δ^15^N_SON_ in Soil Profiles in Karst Critical Zone Observatory (CZO), Southwest China. Sustainability.

[37] Liu M., Han G., Zhang Q. (2020). Effects of agricultural abandonment on soil aggregation, soil organic carbon storage and stabilization: Results from observation in a small karst catchment, Southwest China. Agric. Ecosyst. Environ..

[38] Barbieri M. (2016). The importance of enrichment factor (EF) and geoaccumulation index (I_geo_) to evaluate the soil contamination. J. Geol. Geophys..

[39] Müller G. (1969). Index of geoaccumulation in sediments of the Rhine River. Geol. J..

[40] Hakanson L. (1980). An ecological risk index for aquatic pollution control, a sediment-ecological approach. Water Res..

[41] (1990). China Environmental Monitoring Station: Background Value of Elements in Soils of China.

[42] Zeng F., Ali S., Zhang H., Ouyang Y., Qiu B., Wu F., Zhang G. (2011). The influence of pH and organic matter content in paddy soil on heavy metal availability and their uptake by rice plants. Environ. Pollut..

[43] Lv J., Liu Y., Zhang Z., Dai J., Dai B., Zhu Y. (2015). Identifying the origins and spatial distributions of heavy metals in soils of Ju country (Eastern China) using multivariate and geostatistical approach. J. Soil Sediments.

[44] Chen T., Liu X., Zhu M., Zhao K., Wu J., Xu J., Huang P. (2008). Identification of trace element sources and associated risk assessment in vegetable soils of the urban–rural transitional area of Hangzhou, China. Environ. Pollut..

[45] Dumat C., Quenea K., Bermond A., Toinen S., Benedetti M.F. (2006). Study of the trace metal ion influence on the turnover of soil organic matter in cultivated contaminated soils. Environ. Pollut..

[46] Wang H. (1991). Advances of metal pollution study in soils. Environ. Chem..

[47] Li F., Huang J., Zeng G., Yuan X., Li X., Liang J., Wang X., Tang X., Bai B. (2013). Spatial risk assessment and sources identification of heavy metals in surface sediments from the Dongting Lake, Middle China. J. Geochem. Explor..

[48] Gray C.W., McLaren R.G., Roberts A.H.C., Condron L.M. (1999). The effect of long-term phosphatic fertiliser applications on the amounts and forms of cadmium in soils under pasture in New Zealand. Nutr. Cycl. Agroecosyst..

[49] Fu C., Guo H., Pan J., Qi J., Zhou W. (2009). Potential ecological risk assessment of heavy metal pollution in sediments of the Yangtze River within the Wanzhou section, China. Biol. Trace Elem. Res..

